# Life experienced as worth living and beyond: a qualitative study of the pathways to recovery and flourishing amongst individuals treated for borderline personality disorder

**DOI:** 10.1186/s12888-023-05357-9

**Published:** 2023-11-14

**Authors:** Sophie I. Liljedahl, Anni Mossberg, Hanna Grenner, Margda Waern

**Affiliations:** 1 Region Västra Götaland, Department of Psychiatry for Affective Disorders, Sahlgrenska University Hospital, National Specialized Medical Care Unit for Severe Self-Harm Behaviour, Journalvägen 5, Gothenburg, 416 50 Sweden; 2 Department of Psychiatry and Neurochemistry, Institute of Neuroscience and Physiology, Sahlgrenska Academy, University of Gothenburg, SU/Sahlgrenska, Blå Stråket 15, Gothenburg, 413 45 Sweden; 3 Region Västra Götaland, Sahlgrenska University Hospital, Psychosis Clinic, Gothenburg, 41345 Sweden

**Keywords:** Dialectical Behaviour Therapy (DBT), Borderline personality disorder (BPD), Life worth living, Recovery, Facilitators of recovery, Flourishing, Thematic analysis, Lived experience

## Abstract

**Background:**

Dialectical Behaviour Therapy (DBT) is recognized as a leading evidence-based treatment, effective in reducing symptoms of borderline personality disorder (BPD), as well as co-occurring clinical syndromes. However, symptom remission may not be the same as a life experienced as worth living. The purpose of the study was to understand, from the perspective of individuals with lived experience, the concepts of recovery, life experienced as worth living and flourishing after treatment for BPD, and to describe the pathways to wellness after symptom remission.

**Methods:**

Semi-structured interviews were conducted with nine adult women previously diagnosed with BPD, co-occurring clinical syndromes and severe self-harm behaviour who self-identified as recovered for a minimum of two years, recruited from a network for individuals with lived experience. The average duration of recovery was 5.7 years with a range from 2 to 10 + years. Data were analysed using thematic analysis.

**Results:**

Four main themes and 14 subthemes were generated from our analyses. Main themes indicated that loved ones helped recovery and to create a life worth living, that participants identified as recovered and as healthy and beyond, and that becoming well is a long process associated in part with reclaiming a healthy identity. Participants defined recovery as separate but related to a life worth living, which in turn was separate but related to being healthy and having lives they described as being beyond health and well-being. The wellness process was described as lengthy and non-linear, including setbacks that with time no longer derailed daily life. A proposed theoretical model depicting the wellness process over time from symptom remission to the experience of a life beyond health and wellness is presented.

**Conclusions:**

This qualitative study contributes knowledge of what a life experienced as worth living means, as well as how wellness progressed into flourishing for some participants within a sample of individuals with lived experience. Our findings may inform treatment development that targets more than symptom reduction, which in turn may shorten trajectories from symptom remission to health, wellness, and flourishing.

**Supplementary Information:**

The online version contains supplementary material available at 10.1186/s12888-023-05357-9.

## Background

There is much promise for symptom remission following specialized, evidence-based treatment for borderline personality disorder (BPD) [1]. Dialectical Behavior Therapy (DBT) is a leading specialized, evidence-based treatment for BPD [2]. It is a staged treatment with a hierarchy of therapy targets that prioritizes staying alive, staying in therapy, improving life quality and skillful responding as the targets of “Standard DBT” (Stage 1). DBT then progresses to Stage 2, which is concerned with addressing any outstanding mental illness, as multiple co-occurring clinical syndromes are common with a diagnosis of BPD [3], particularly co-occurring posttraumatic stress disorder (PTSD) [4]. Stage 3 is concerned with creating a life with normal ups and downs, in which meaningful goals are pursued and self-worth is increased. Stage 4 is dedicated to resolving any sense of incompleteness, for example connectivity to a sense of meaning and purpose via spirituality for those who seek [5]. Finding freedom and fulfillment in life in Stage 3 or 4 is defined as having a life experienced as worth living or “life worth living” in short [3, 5].

The process of testing innovations in psychotherapy via randomized controlled trials (RCT) to determine efficacy and effectiveness [6], and to establish DBT as an empirically based treatment was a necessary but lengthy process [1]. A limitation of RCT research is that the standard outcomes tend to focus on symptom remission. Given the need to continue to test and develop the effect of DBT in reducing suffering amongst complex populations [7, 8] in novel treatment delivery formats [9, 10] it is only recently that the research community has understood that symptom remission is not the same as recovery [11, 12]. Research on recovery beyond symptom remission suggests this is a currently unmet need, particularly in relation to qualitative studies that involve the perspectives of those with lived experience of BPD in relation to DBT treatment [13].

There is a limited amount of research on definitions of life-worth-living and related recovery goals in the DBT literature to date. The DBT-accepting the challenges of employment and self-sufficiency (DBT-ACES) program explicitly focuses on conceptualizing recovery goals collaboratively (individual and therapist together) while supporting individuals to engage in work or continuing education with support [14, 15]. DBT-ACES may be offered following one year of standard DBT to support individuals’ efforts towards employment and self-reliance, and termination of psychiatric disability (financing) as well as exiting the mental health system [15]. The authors of the DBT-ACES feasibility trial [15] quote Barak Obama’s New Freedom Commission for Mental Health [16] in defining recovery as “The process in which people are able to live, work, learn, and participate fully in their communities. For some individuals, recovery is the ability to live a fulfilling and productive life despite a disability. For others, recovery implies the reduction or complete remission of symptoms”.

Research dedicated to the study of recovery amongst individuals diagnosed with BPD from the perspective of lived experiences suggests that treatment can pose both barriers and facilitators to becoming well. In their qualitative analysis of perspectives from individuals receiving DBT and DBT-ACES, Carmel et al. [17] evaluated individuals’ perceptions of how their problematic behaviour was reinforced by the treatment providers and loved ones in the context of their environment. Content analysis of participant responses in Carmel et al.’s study suggested that behaviours not aligned with recovery that was reinforced by others were: (1) not engaging with demands of life and ongoing treatment (avoidance), (2) suggestions of coping behaviours that were not effective or aligned with goals, and (3) expression of low expectations with respect to individuals’ capacity [17]. Often these communications were benignly intended but unhelpful, nevertheless. Carmel et al. named stigma as a “central barrier to recovery” as described by the individuals in their study [17]. They further described recovery as a psychosocial process. While the authors focused upon barriers to recovery in their work, they suggested follow-up studies that evaluate factors facilitating recovery [17]. Herein lies the purpose of our research, alongside a desire to conceptualize if and how a life worth living and flourishing may be attainable for some individuals as part of or following recovery. Flourishing refers to optimal mental health whereby a person possesses and demonstrates high levels of psychological well-being, emotional health, and social connectivity [18, 19].

The overall aim of the study is to capture the lived experience of individuals with a former history of BPD and self-harm with respect to the process of recovery beyond symptom remission, with a focus on defining a life worth living and flourishing. We further aimed to determine at what point in their lives individuals who self-identify as “recovered” stop identifying as “patients”. This study is part of an ongoing three-site collaboration project dedicated to understanding the experience of individuals diagnosed with BPD who are (a) currently in standard outpatient DBT treatment, (b) receiving intensified inpatient DBT, or (c) self-identify as recovered, with histories of treatment for BPD including DBT. The current study is focused on the recovered cohort.

## Methods

### Sample

A total of nine participants completed study interviews. The number of participants was determined to be sufficient to develop clearly defined themes within qualitative thematic analysis, as the questions asked using a semi-structured interview schedule were related to concepts and experiences that participants were knowledgeable about from their lived experience [20].

Recruitment procedures are described below. All participants identified as female. The median age at the time of the interview was 32 years (with a range from 24 to 41 years). With respect to sexual identity, the majority (six) identified as bisexual, two as homosexual and one as heterosexual. Most participants (seven) lived in large cities, with their partner (six). Two lived with children for whom they were responsible, and one lived with children for whom they were not responsible. Just over half the sample (five) were employed as their primary source of income, while four had student loans as their primary source of income. The majority of participants had university education (seven), while one was pursuing a high school diploma and one had mandatory schooling only (completed grade 8). All participants identified as being of European ethnicity and all interviews were conducted in Swedish. With respect to previous diagnoses conferred, all participants endorsed having been diagnosed with borderline personality disorder (BPD), and co-occurring disorders including mood disorders (n = 6), anxiety disorder (n = 5), eating disorder (n = 4), posttraumatic stress disorder (n = 3), attention deficit hyperactivity disorder (n = 2), and substance use disorder (n = 1). Historically conferred diagnoses in relation to mental illness were derived from participant self-report on a Background Questionnaire querying demographics and mental health history.

Just under half of the study participants (four) had ongoing pharmacotherapy, with three participants taking medication for mood and/or anxiety, attention or sleeping difficulties and one taking an opiate antagonist along with sleep and anxiolytic medication. With respect to self-definition as recovered, duration of recovery ranged from two years to more than 10 years (average 5.7 years) at the time of interview.

### Measures

A background questionnaire was created by the first author to query demographic characteristics, historically conferred diagnoses, and ongoing pharmacotherapy. Interviews querying recovery, life worth living and beyond followed a semi-structured and open-ended interview guide (please see supplementary File 1) constructed for this study and piloted with individuals with lived experience employed in the first author’s hospital setting. The purpose of open-ended questions for participants was to encourage their formulation of key constructs under study such as recovery and a life worth living, without prompting or influencing their ideas or responses. At the end of the interview participants were asked: *“Is there anything else I have not asked you about that you think is important to add?”* In accordance with Braun and Clarke’s [20] recommendation of flexibility and revisiting protocols as new knowledge is gathered, two additional questions regarding identity were added after the third participant indicated that identity was a significant aspect of both her illness and her wellness (Please see Supplementary File 1).

### Procedure

***Recruitment***. Individuals who self-identify as “recovered” for a minimum of two years from BPD or PTSD or both as well as longstanding difficulties with self-harm and suicidality were recruited from Sweden’s self-harm and eating disorder network (SHEDO), a non-governmental, non-profit organization for individuals with lived experience or interest therein. A flyer with information about the study was sent by the first author to two SHEDO representatives who distributed the information within their networks and on social media. Information about the study was also shared through word of mouth. The flyer included contact information for the first author and an invitation to be in touch for those interested in study participation. All materials and procedures were approved by the Swedish Ethical Review Authority (Diary Number: 2020–03139).

Following initial contact with the first author to determine eligibility for participation, information letters about the study and the nature of participation along with consent forms and a demographic background information form were sent by mail to prospective participants. Once informed consent was obtained a telephone interview was scheduled.

***Inclusion criteria***. Individuals were invited for study participation based on the following characteristics and experiences: (1) History of severe and repetitive self-harming and life-threatening behavior; (2) Historic principal diagnosis of BPD or BPD and PTSD; (3) Treatment with DBT; (4) Self-identified as recovered for a minimum of two years; (5) Age between 18 and 65; (6) Ability to give consent; (7) Ability to speak Swedish or English. In this study of lived experiences, operational measures of psychopathology, treatment duration and adherence were not available, nor were they deemed necessary in the context of qualitative inquiry that defers to individual narrative history.

***Exclusion criteria***: There were no exclusion criteria for this study.

A total of 11 individuals indicated interest in study participation, met inclusion criteria and were mailed consent forms and background questionnaires in return envelopes. Two of the 11 prospective participants returned their completed background questionnaires without consent forms or a return address on the envelope posted, making it impossible to determine to whom they belonged; therefore, nine participants comprised the final convenience sample.

### Data collection

The first author conducted interviews with study participants between November 2020 and October 2021. The average duration of interviews was 53 min (range of 30–78 min). Interviews were conducted by telephone to facilitate participation from eligible individuals nationally, and due of the COVID-19 pandemic. Interviews were audio recorded. Transcription was completed by the third author and translation to English was completed by the first author, with back-translation between both authors in instances of ambiguity and uncertainty. Possibly identifying information was removed.

### Data analysis

In this qualitative study, thematic analysis was selected for data analysis because of its versatility that allowed an explorative investigation of the interview material [21]. To answer our scientific questions, a total of 9 participants were recruited for study participation. Nine participants were deemed be sufficient to develop clearly defined themes within qualitative thematic analysis with sufficient depth, since the questions asked with the interview schedule are concepts and experiences that participants were knowledgeable about from their lived experience [21].

The analysis was approached from a critical realist epistemological position, assuming an ontological reality [22], which was of scientific interest through the accounts of the participants, while simultaneously recognizing that sociocultural processes shape our understanding and meaning making of the world [23]. Interpretation of the data was conducted at the semantic level. Further, a mixed inductive (bottom-up) and deductive (top-down) analysis was employed [24] where the data codes were derived from the experiences of the participants. Theories relating to BPD [3, 5], flourishing in the context of well-being [18, 19] and research on recovery amongst individuals with BPD receiving DBT treatment [14–15; 17] was drawn upon when formulating themes in data analysis.

The thematic analysis began at the end of the data collection period, following the six phases outlined by Braun and Clarke [25]: familiarization; coding; generating initial themes; developing and reviewing themes; refining, defining, and naming themes; writing up. The first and second and fourth author began familiarizing themselves with the material by reading and re-reading the transcribed interviews while making initial notes. The data were then systematically double-coded by two separate coders labeling units of meaning. The software programme NVivo 12 was used by one coder and NVivo 1.6.1 was used by another^1^. The first author coded the data independently in consultation with the third author who was familiarized with the material from the transcription process and had previous experience of qualitative investigation. The second author received consultation from the fourth author who was also familiarized with the data and knowledgeable about thematic analysis. Good convergence in the coded material was obtained in a group meeting between both coding teams who shared their coded datasets (one per team and two in total). At this time, we observed multiple overarching commonalities, with negligible differences. All authors discussed the themes. This process had an iterative nature which involved returning to the interview material to identify data extracts which simultaneously functioned as a way of ensuring the validity of the themes, consistent with Braun and Clarke’s [25] recommendations. The involvement of multiple analysts with differing theoretical orientations^2^ served as a way of deepening the understanding of the material and triangulation of the findings, as well as stimulating reflexivity. Due to the magnitude of the material, which had been collected and coded within the larger research project, we chose to only include the data codes that were of relevance to the aims of the current paper. This is in line with Braun and Clarke’s [25] guidelines. However, careful efforts were made to not exclude material in a way that would violate the nuance, depth, and unavoidable contrasts within the findings.

### Reflexivity

**Fig. 1:**
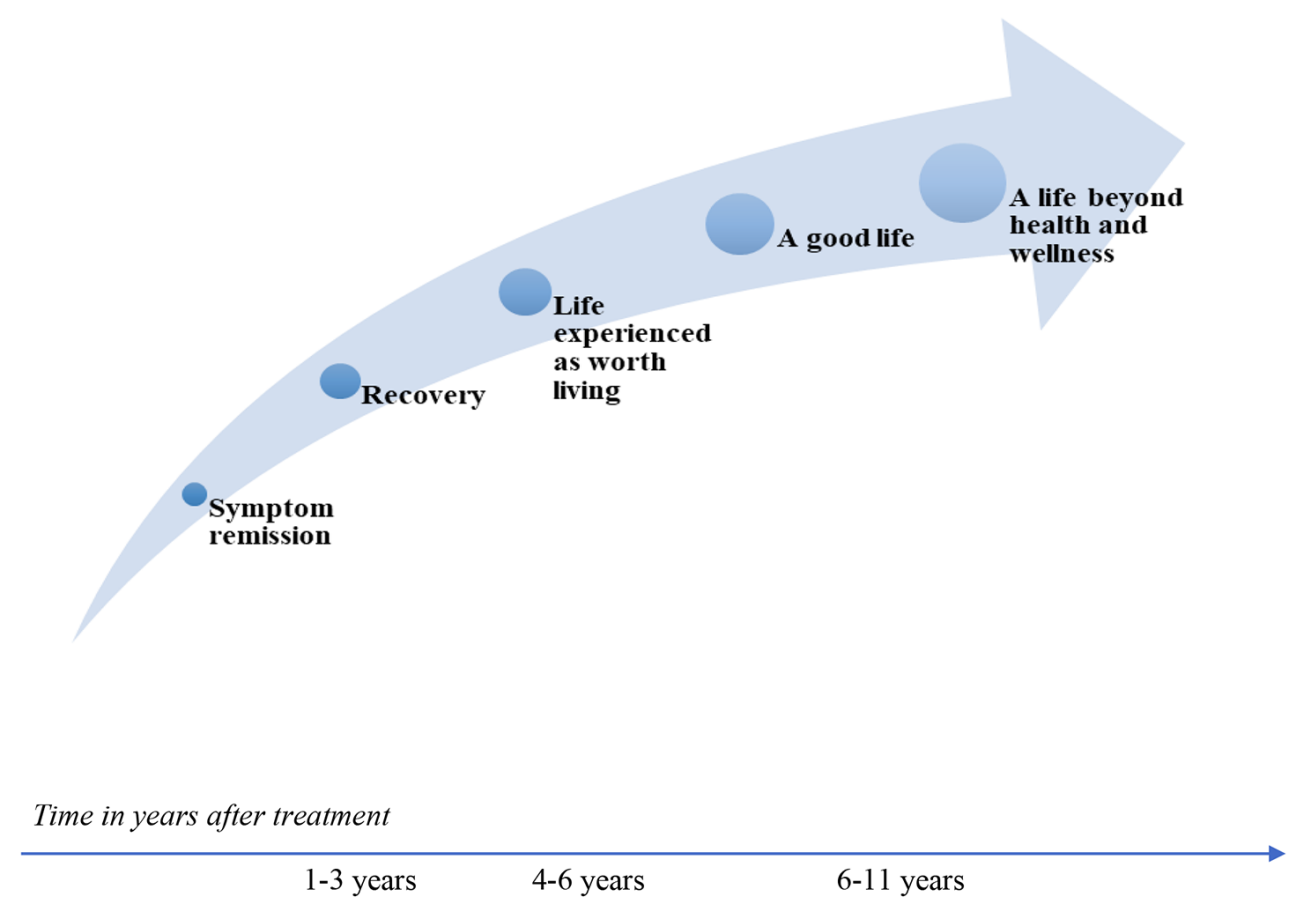
Process from symptom remission to the experience of a life beyond health and wellness

Throughout the study process the authors reflected on and discussed their assumptions and experiences about the illness-to-wellness process generated by interview data as well as by clinical recollections of individuals with similar histories to the study participants. While formulating the proposed theoretical model reflecting the process from symptom remission to health and beyond (Fig. 1) the first author wondered:

*“Am I presenting these individuals as faring better than they may be in reality?”.* Rather quickly came a further reflection *“How often do we wonder whether we are presenting people receiving therapy as faring worse than they may be in reality?”* or perhaps more fairly: *“Given the identity instability of many individuals diagnosed with BPD, should we be more careful to present a balanced view of people in all aspects of our contact with them, including diagnostics and research?*

## Results

Four main themes and 14 sub-themes were derived from thematic analyses (Table 1) describing participants’ experience of recovery and a life worth living (LWL), identification as recovered, identification as being healthy and beyond, and that wellness is a long process. A proposed theoretical model depicting time and process from symptom remission to the experience of a life beyond health and wellness was also generated from interview data (see Fig. 1; Table 2). The order of themes presented follows the temporal sequence in which participants described their processes of recovery, a life worth living, and beyond. Table 2 anchors these processes to years post-treatment as described by study participants.

**Table 1:** Themes and sub-themes describing participants’ experience of symptom remission, recovery, and a life worth living, being beyond healthy, and becoming well

Themes	Sub-themes
Family or close people helped recovery and to create a LWL	Family or close people helped recovery
	Family or close people helped creating a LWL
Identify as recovered	Is almost recovered (has LWL) LWL is not the same as being recovered LWL needs to have depth; it’s not about recovery You must at least be partly recovered to have a LWL Being recovered is not a static condition
Identify as healthy and beyond	Feels far from mental illness
	Inner work to develop strength and self-belief
	Cultivated wisdom through meaning and perspective
	Life beyond dreams
Becoming well is a long process	Took many years to become more healthy than sick Intensive multi-year therapies necessary Too short, non-adapted or incomplete treatments not helpful

### Theme 1: family or close people helped recovery and to create a LWL

This theme reflects the centrality of having relationships with loved ones to support the journey of becoming well. All nine participants spoke about the importance of having closeness, both for helping stabilize recovery in its early stages and to create a life worth living. For some participants, loved ones were described as giving strength, courage, and internal fortitude to continue the hard work of therapy when they felt demoralized or at their worst. Others described the mere presence of families and loved ones as healing.

In addition to the vitality of relationships with loved ones, participants also discussed the healing effects of caring for animals that were perceived as easier to be with than people at times.

### Family or close people helped recovery

This subtheme highlighted how family and loved ones helped participants to maintain and stabilize their recovery in the early stages when new healthy behavioral patterns were still becoming familiar. One participant reflected the essence of this subtheme:

*“…in the beginning it was kind of a lot that I felt I wanted to do it* [stay free from self-harm] *for my boyfriend…Because we moved in together and then it was, it was a good thing, like, having someone close.”* (P8).

In sum, participants noted that small changes became important to sustaining big changes in relation to patterned behavioral problems such as self-harm and eating disorders, and that the presence of loved ones helped them stabilized early recovery gains.

### Family or close people helped creating a LWL

This subtheme related to participants’ sense of how their family and loved ones contributed not only to their ability to *attain* a LWL but also to their ability to *create* a LWL, which was described by most participants as the step that followed recovery. Regarding the experience of being with loved ones within the context of identifying as recovered and having a life worth living after many years of somatic illness and psychiatric hospitalization one participant reflected:

*“…* [I] *dare to be with my loved ones fully… to have the ability to thus maintain good and close relationships. That, it gives me such, like, a rush of happiness, when I am with people and feel that, that I can be in this nice and glorious* [place in my life] *and I am not afraid that it will end, and I will experience it again…”* (P5).

Many participants described finding self-acceptance and then self-value through the recognition that they were lovable and loved by family or close people. Being loved and loving in return connected several participants to the possibility that their future lives, free from illness, could be different from their experience of themselves with illness in the past.

### Theme 2: identify as recovered

Many participants defined recovery in relation to completing therapy for their principal diagnosis and no longer self-harming. This main theme was comprised by participants’ descriptions of how they viewed the concept of recovery based on their lived experience. Participants both reflected on their past recovery process in relation to their current life today, as well as the utility of the term “recovery” itself. Although all participants self-identified as recovered at the time of study inclusion, three preferred other terms during the research interview.

### Is almost recovered (has LWL)

Participants described recovery and LWL as processes that coalesced. This subtheme is comprised of descriptions regarding how participants formulated recovery and LWL as distinct processes. One participant who identified as mostly recovered said that she had a LWL but was not yet completely recovered:

*“I probably do not want to say 100% recovered but I still think I am very close. So, if I am going to take it as a percentage, I probably actually want to say that I am enough, aah, 90% recovered.”* (P2). For this participant having a “taste” of a LWL during the relatively early stages of her recovery was necessary for her to persist in difficult behavioral change including giving up substance use and self-harming behaviors.

### LWL is not the same as being recovered

Of those who identified as recovered, the majority (six out of seven) reported feeling that recovery was not the same as a LWL. On a semantic level this subtheme was generated by participants’ observations of meaningful differences whereby recovery was associated with symptom remission and a life worth living was associated with engaging with life and experiencing its value:

*“That you are recovered is like more of a zero position, the lack of negative things. But what I think* [is] *that a life worth living can be very many different things, but I think there you are at least a plus”.* (P7).

Some of our participants reflected that for them life was always worth living, but that once recovered having a LWL flowed more readily and easily than experiencing LWL with limited resources while ill.

### LWL needs to have depth; it’s not about recovery

This subtheme was comprised by participants contrasting similarities and differences between the terms of “recovery” and a “LWL”, as reflected upon by their experiences of both. One participant reflected upon the process of moving past the sense that life was (only) worth living in relation to the meaning she experienced in her life today. She recalled her experience of recovery in similar terms, with respect to depth:

*“…for me to think that life is worth living, it needs to have a certain depth…I imagine that I could experience myself as recovered…and still have a life that is quite flat. And I imagine that…my recovery has been a process that is part of a larger process of creating a life that is worth living but* [recovery] *is only a part.”* (P4).

### You must be at least partly recovered to have a LWL

This subtheme is comprised by participant reflections that recovery and a LWL are necessarily linked with the requirement of having some degree of recovery prior to having a LWL. One participant described her experience as feeling that recovery and a life worth living were overlapping processes (see Fig. 1):

*“I think* [they go] *together. Because I think if I had not been given a life worth living, it would have been difficult to recover. It kind of hangs together. Because without a life worth living, I probably would still have felt bad. And then I would not have recovered. Then maybe there are different degrees of life worth living, that is, that you get to where you feel you have almost everything, so it is not difficult, it is just small difficulties…”.* (P2).

### Being recovered is not a static condition

Three participants described their current functioning as healthy and beyond recovery or a LWL (see Theme 3 below). Accordingly, they did not identify their current state as one captured by either of these terms, but had reflections about these states and experiences, encompassed within this subtheme:

*“I definitely see myself as a person who has recovered from mental illness and so on. But… I do not see mental illness as a static condition, so…I do not think it is so relevant to talk about it from the aspect of whether you have been diagnosed or what symptoms you have…. That is not what is interesting to me. …When I look from the inside, I see myself as, uh, more resilient. So, I would describe as well as my recovery, as that I have more resilience to face what life gives me, whether it is mental illness or other challenges in life.”* (P3).

These perspectives of recovery and a life worth living were directly related to how participants identified as living today and in contrast to their history of illness and process of becoming well. A key aspect of this subtheme is how things changed with respect to how they identified based on where they were in their wellness process. Participants reflected that they found their healthy identities through pursuing activities that they found exciting or fun, and in relation to others, and particularly when they felt that their presence made a positive impact to others.

### Theme 3: identify as healthy and beyond

This main theme captures participant descriptions of moving past the experience of identifying with the terms of “recovery” and “a life worth living” into experiences that they formulated as being characterized by health, meaning, and depth. These participants described cultivating inner resources such as self-belief and wisdom and living lives that exceeded their dreams when they were ill. Three participants identified as living lives characterized principally by these descriptors, while the remainder of participants identified with being recovered, having LWL and having good lives (Theme 2).

### Feels far from mental Illness

This subtheme is comprised of participant experiences of feeling experientially separate in their current lives from when their lives were historically characterized by having been ill. In this sense participants described having “moved on” from the period of their lives in which they were principally occupied by having serious mental illness. As one participant observed:

*“I see myself as healthy. Completely…It means that I am no longer in any way, maybe an exaggeration, but controlled by feeling bad. That I am not governed by anxiety, that I am not governed by anything else that bothers me in either mood or what I do or do not do, what I eat or do not eat or anything like that, knowing that I can have a bad day but it’s a bad day, I do not have to hurt myself for it, I do not have to act on it…”* (P7).

The experiential distance from years of life consumed by suffering was a defining feature of this subtheme, as was the sense that current high functioning was stable and enduring.

### Inner work to develop strength and self-belief

This subtheme was comprised by descriptions of *how* participants who identified with being healthy and beyond recovery or a LWL in terms of their current functioning attained this positive state of well-being. One participant identified the fact that it took a particular tenacious strength to have an eating disorder and to repeatedly harm oneself. She noted that the possession of that strength could be an attribute if redirected towards positive goals such as health and well-being rather than eating disordered and self-harm behavior. Along with strength, self-belief was a core aspect of this subtheme describing how participants attained their current high functioning. Self-belief was the key driving force identified by a participant in relation to her ability to return to her studies and catch up the required coursework to become a physician:

*“But then when I started to get well, I realized that I might actually have to invest in that dream and catch up on those subjects I never* [completed] *in high school and actually try. So* [medical studies are] *what I’m doing now. I think it was mostly my, ehm…self-belief. I believed so much more in myself that I believed that I actually could do it.”* (P7).

In sum, doing the inner work to re-target one’s strength that had been previously used to maintain self-destructive behaviors as well as developing and acting in self-belief comprised this subtheme.

### Cultivating wisdom through meaning and perspective

The subtheme of cultivating wisdom through meaning and perspective was an experience raised by the majority (seven) of participants. When discussing the happiest moments of their lives in relation to what makes life worth living and beyond, participants mentioned hallmark moments like getting married and graduation. However, just as often they described having cultivated the perspective of recognizing the importance of everyday happiness. One participant raised the importance to her of having freedom to choose how she lives as essential to recovering years of identity development she lost over the duration of her illness. The absence of this freedom during her illness gave her the perspective of valuing it now:

*“And, uh, because I missed so much, I missed, like, big parts of my teens and big parts of my 20’s. So, so I missed, like, maybe this expansive phase of life where you discover a lot and think about who you are in relation to the world and so on. And there I think that I, that is, that I have no problem making my own choices in that I have been outside, so I have, like, some life choices* [in which I like to choose] *a little different about how I choose to live, like for example that I’m vegan and stuff like that… Now I’m kind of a little eager to see there are other ways to live and what could I learn from it”.* (P3).

Given that participant’s long-term somatic and then mental illness, both of which resulted in years of institutionalization, achieving these milestones and rejoining society and the workforce was extraordinary in a way that allowed her to particularly savour the contrast.

### Life beyond dreams

The three participants who identified as healthy beyond recovery and LWL described having lives today that were beyond what they ever imagined for themselves when they were ill. This was true with respect to capacity for emotional experiencing as well as having the ability to create life in accordance with how one wants to live their experience now and in the future.

“[There is a] *huge difference in what emotional availability I have in my own life. And that means that I have a lot of opportunity to stop in the moment. Eh, and think that my life is beautiful or that life is beautiful, so. That is, that I stop in nature…or when I am with my child and such and we have as an expression of mine, my child can say like this `now you have tears like this again of joy.` And it has been my life, that I can really think that, that, that it is fantastically nice and beautiful, what I get to be part of*.” (P3).

Another participant spoke of deliberately choosing her work and activities to maximize the meaning and magic it brings to her life. In sum, broadened emotional capacity that facilitated experiential presence, as well as the ability to realize living in accordance with one’s preferred conditions were the core components of this subtheme.

### Theme 4: becoming well is a long process

Participants in our sample had long illness histories. Similarly, the process of becoming well was described by nearly all (eight of nine) participants as being long and not necessarily linear. Participants observed that becoming well required cultivating not only skillful behaviors through therapy but also the ability to have faith in the world and humanity. After experiencing invalidating family and care providing environments, being able to trust again was described a gradual but essential process in becoming well. Similarly, therapies that were intensive and long-term, and sometimes in consecutive or repeated rounds was described as necessary. Insufficient and non-adapted treatments were associated with backsliding, chronicity and death via observation of others with lived experience.

### Took many years to become more healthy than sick

This subtheme was comprised by participants describing what they did in long-term therapy, explaining why it was necessary for them to become completely well. One participant described the time and effort it took for her and her care providers to restore her ability to take the risk that others would not harm her:

*“So it has taken a very long time to rebuild a feeling that the world, that is, - not naive but still with some kind of nuance – that the world is pretty good and that others want me quite well and such. That at least there are some who want me well and that there are nice things in life, has taken me a long time to, to feel… I worked hugely hugely hugely, very long and a lot. Not just me, my, my poor psychologists and therapists too… to dare to trust”.* (P.3).

A core aspect of this subtheme is that it can, for some individuals, take years to view the world from a new perspective that is aligned with beliefs that support mental health and positive well-being.

### Intensive multi-year therapies necessary

Participants noted that multiple intensive therapies were sometimes also necessary to become completely well, and that the process of understanding oneself came when it was ready, which was not necessarily within prescribed treatment durations based on specific protocols. The necessity of intensive and sometimes multi-year therapies to contribute to the process of becoming well was the core of this subtheme. One participant described this as follows:

*“Ah, but I think one, an important difference has been that like, so all the help I have received, therapy and help to sort of process things and such and I have received it in lots and lots of rounds and certain periods…partly that I had to go so long I think made a very big difference. And that it was so regular, it was, like, many hours in total….when I have sought help I have always, I have thought that I have been completely honest and shared all that I think needs to come out…I have, like, never consciously withheld anything* [due to] *not trust*[ing] *the person or anything like that. But in this therapy so, there were some things that did not emerge until maybe one, one-and-a-half years into the therapy…So there was probably a value in it being over, over a longer period of time…I experienced it as if it was more about like, a little so dare to meet myself.”* (P4).

### Too short, non-adapted or incomplete treatments not helpful

The final subtheme comprised in Main Theme 4 related to consequences of treatments not aligned with patient needs and circumstances. Consequences were described in relation to losses for participants and others with lived experience either in terms of clinical worsening, losing years of life experienced as not worth living, and death of friends with similar mental health issues who were not able to recover. As reflected by a participant regarding the harm of too short, non-adapted or incomplete therapies:

*“I am very grateful that I got to be* [in a treatment home] *for so long because it really made me have all the tools I needed to cope afterwards. Because…things happen…and had I not had treatment for so long, I’m not sure I would have made it through those setbacks… Because so many times I saw it in the treatment home that some did not get the care time required which made you become recurrent and become not finished and some survive as well as not, either… And I don’t think that I’m unique in a way just because I have gotten out of it. I think it is very very important to give the person the opportunity to actually be completely done.”* (P6).

This participants’ reflections suggest that by granting others the same conditions she experienced to become well that chronicity and death may be reduced in individuals with similar mental health histories and needs.

**Table 2:** Experiences of recovery, life worth living, and being healthy and beyond linked to transcribed interview data

*Participant #*	*Years self-identified as recovered*	*How participant identifies and experiences life today*	*page # interview transcription*
1	8	Identifies as “recovered”; “has a life worth living”; “a good life”; life “exciting” “fun” “beautiful”	1; 2; 4
2	3	Feels “90% recovered”; “I want to live and that’s why it’s so good to say that it’s a life worth living.” Life today feels like “a valuable gift that…means a lot to have received.”	1; 4; 5
3	3	Objectively “recovered” but prefers to view herself as resilient; has life worth living; Life “exciting” “fun” “free”. “Sometimes I stop and think that I am lucky and that I have such a fantastic life”	1; 3–4, 6; 8
4	8–10	Has recovered. Has a life worth living - feels meaningful; life can be beautiful and in periods “absolutely extraordinary magical”	1; 4; 7–8
5	5–6	Is recovered. “[Life] as it is now I could not even dream that it would be”. Life “absolutely” worth living. “Adversity… is always [there] but does not overthrow me anymore”. “Resilience”	1; 2; 3
6	5	Fully and completely recovered. Life worth living “this is a life I wanted for very many years” “…it is very beautiful to be able to take part in different parts of life.”	1; 2; 3
7	10–11	“Recovered and Healthy. Completely.” “I live a life worth living and that is meaningful. I, in my studies, I am somehow on my way to fulfilling a dream I have had for a very, very long time”	1; 2
8	5 in total, 2 following a difficult year	Identifies as “recovered”; life “mostly” worth living and meaningful. “Life is fun”; “in short moments Beautiful”	1; 3
9	5	“I do not think of myself as recovered, rather as … healthy. [I] feel very far from … borderline and stuff, sometimes I almost forget it. “Absolutely experience” life as worth living. “It is very wonderful to be able to go out into the world”	1; 2; 3

Figure 1 depicts the time and process from symptom remission to the experience of a healthy life and beyond as described by study participants. To anchor the wellness process depicted in Fig. 1 to participants’ current functioning at the time of the interview, Table 2 was developed, which reports the participant number, the duration of years self-identified as recovered, and quotes related to how they currently experience their lives. Time in years is depicted in an arrow at the base of the figure, running left to right, with time in years beginning at symptom remission to the time of interview.

Of note in Fig. 1 is the non-linear nature of becoming well. Some participants described themselves as being recovered, having a life worth living, and being healthy whereas others no longer identified with recovery as they had moved beyond that self-definition towards life characterized by health and beyond health and wellness. One participant (P8) had had a significant setback after feeling well for three years. Although she did not return to self-harming behaviors or full-blown illness, she did not yet view her life as beyond health and wellness, and also expressed concern over whether she ever would. Of those participants who identified in terms of living a good life, health and beyond health and wellness they were all recovered for more than four years (P5, P7, P9). Participant 4 endorsed aspects of being beyond health and wellness in her life at the time of the interview, but also described ongoing vulnerability to seasonal affective disorder and the need to manage attention deficit hyper-activity disorder (ADHD) which she described as akin to an allergy. Her functioning fluctuated between beyond health and wellness at times of feeling good and recovery/having a life worth living at times when she would be required to work hard at using skills she developed in treatment. The same is true for P3 who described her life in terms of being beyond health and wellness at times, but also in terms of recovery when she felt vulnerable or in need of protecting her wellness and energy. Notably, participant 3 had only been recovered for three years.

Finally, none of the participants identified as being “patients”, which was described as being left behind with “sickness identities” somewhere after symptom remission and the first one-to-three years post-treatment after which time those without major setbacks or ongoing vulnerabilities stepped more fully into recovery, a life worth living, a good life and a life characterized by a life beyond health and wellness (Fig. 1; Table 2).

## Discussion

Thematic analysis of interview data from nine recovered adult women generated four main themes in relation to how loved ones helped participants to sustain recovery and create a life worth living, identification of oneself as recovered, identification of oneself as healthy and beyond and that becoming well was a long process. Subthemes encompassed by overarching themes provided specific insight into our research aims. Most individuals felt that recovery and a life worth living were not the same; that recovery preceded a life worth living, and that a person reached recovery after symptom remission but before achieving a life worth living. Our findings are consistent with the position of the DBT literature wherein it is recognized that symptom remission may be the beginning of a recovery process, but that it is not the same as recovery [11, 12]. A good life and life characterized by health and current functioning beyond health and well-being was described by a portion of participants who identified with these descriptors, whose recovery had been longer term (4 + years). They described achieving lifelong dreams, for example, of studying to achieve one’s dream job, of thriving in life that felt full of possibility and wonder, and of feeling lucky to have lives they felt were rich and meaningful. Participants also remarked upon the normalcy and expectation of having bad days, challenges, and adversity as necessary components of living well. However, participants described now having the skills and resources, perspective, strength, and self-belief to meet these challenges without becoming derailed in their current lives and states of well-being.

Experiences of recovery described by our study participants are congruent with the definition of recovery reported by Comtois et al. [15], inspired by quote from Barak Obama expressed 20 years ago in the United States of America [16]. Recovery was characterized by full community participation, living fulfilling lives despite ongoing struggles in for some individuals, while complete freedom from symptoms would be the recovery experiences of others [16]. Findings from our thematic analyses suggest that complete longer-term treatment is associated with the experience of being recovered, and that identifying as recovered is a significant step towards experiencing life as worth living, but not the same as a life worth living. Identifying with having a life worth living was in turn associated with having a good life and achieving a life beyond health and wellness, with each stage coming at longer duration of follow-up for participants in our convenience sample. These findings were anchored by number of years recovered within a proposed theoretical model depicting the wellness process from symptom remission to the experience of life beyond health and well-being.

As introduced earlier, flourishing refers to states of ideal or optimal mental health in which individuals report consistent and stable psychological well-being, good emotional health and sustaining social connectivity [14, 15]. The final stage in the process of becoming well (Fig. 1), referred to in Theme 3 as *Identifying as healthy and beyond health and wellness* is consistent with this definition of flourishing. In other words, 3 of the study participants´ current functioning would be consistent with flourishing, and 2 participants would meet the definition of flourishing in periods of greater well-being fluctuating back to having recovery and a life experienced as worth living in times of greater vulnerability. Participants who had stably flourishing lives identified as having 4–6 years of recovery whereas those who had periods of flourishing had as little as 3 years of recovery at the time of interview.

With respect to recovery and a life worth living, participants expressed experiences and conditions that are normal by Swedish standards. Savouring moments in nature, with loved ones, of fun and of life’s beauty and magic were associated with recognition that recovery had progressed sufficiently that one’s entire life and focus were not consumed by feeling horrible all the time within the process of becoming well. Many participants described the relief of recovery that allowed energy to be restored for more than the experience of feeling badly. It was at this point that they described regaining curiosity in others and in life and the role of “external factors” such as interests, romantic partners, families, hobbies, fun, meaningful or fun work and engagement. Connecting with these “external factors” once recovered was the transaction that defined the experience of a life worth living.

We propose that the “external factors” described by our study participants in relation to their recovery, lives worth living and in some cases, flourishing may be the facilitators of recovery called for by Carmel et al. [17] in their study of barriers to recovery, as recommendations to evaluate facilitators of recovery in future research. The first and most enduring “external factor”/facilitator named by participants when recalling their pathways to becoming well over time was connectivity to loved ones. Participants’ ability to identify themselves as worthy of love and capable of enhancing others’ lives by their presence, and then finally trusting that others would stay were themes that were interwoven over wellness trajectories for our study participants. In this way, our findings are aligned with earlier studies of recovery amongst individuals diagnosed with BPD with experience of DBT in the classification of recovery as “psychosocial” in nature [14–15; 17]. Other “external factors”/facilitators of wellness described by study participants were cultivating interests and hobbies, having fun, and being engaged in meaningful work including continuing education. The latter two initiatives are encompassed by the DBT-ACES program, which was developed to support recovery, self-reliance and exiting the mental health system [14–16]. Internal facilitators of life worth living and beyond that emerged in our findings as critical to becoming well were: Daring to trust in oneself and others; self-belief; resilience; “tenacious strength” refocused from self-harm and eating disorder behaviours to building a life that was previously unfathomable; and wisdom. Linehan alludes to some of these characteristics, such as wisdom, in her description of Stage 4/ life experienced as worth living in her most recent treatment manual [5]. However, to the best of our knowledge at the time of writing, these aspects of a life worth living and beyond have not been previously reported from individuals with lived experience in relation to their lives after treatment.

With respect to identity, the experience of being a “patient” was tied to being in treatment up to the point of symptom remission, and no longer characteristic of current functioning for any participant at the time of the study interview. A number of participants reported that reclaiming their identity as recovered and healthy mirrored their wellness process, as they noted that having a “sickness identity” was associated with doing poorly and feeling badly, often in equal magnitude. The fear of not knowing who they were if not sick was expressed, as was the strength necessary to dare to trust in the wellness process that at one point was unknown terrain.

Finally, participants reported that becoming well was a lengthy and not necessarily linear process. The role of completing a sufficiently long treatment adapted to their needs was expressed as essential to achieving symptom remission as a first step on the road to recovery and beyond. Notably, four study participants were taking maintenance doses of pharmacotherapy.

Treatment adaptation was proposed to be a key method of effectively implementing treatments for complex needs; failure to adapt treatments to individual needs was described as harmful, reminiscent of Carmel et al’s [17] report of stigma as a barrier to recovery in this population. Our findings suggest that becoming well from symptom remission to flourishing is a trajectory that is more likely if individuals receive adapted, longer-term and complete therapies than shorter, non-adapted or incomplete treatments. An example of an adapted treatment could be adding an evidence-based eating disorder protocol to one’s DBT treatment hierarchy (adapted), rather than telling individuals that they could not be eating disordered and receive treatment in a particular setting (non-adapted). Completed long-term therapies were also associated with having sufficient skills to manage setbacks, slips and periods of adversity, which took less and less time to “bounce back” from the longer that individuals identified as recovered. Adapting DBT via mixed inpatient and outpatient care to meet the needs of individuals with severe BPD presentation is a recent treatment development [10] consistent with the needs expressed by our participants.

There may be a new movement towards evaluating flourishing, thriving and optimal mental health after treatment [26]. Similar to our view, Devendorf et al. observe that long-term follow-up studies of well-being amongst individuals receiving treatment for mental illness are scarce. Devendorf et al. [26] focused upon optimal well-being (OWB) in a population-based sample of individuals treated for mood and anxiety disorders, suicidality, and substance use disorders and used a scaled definition of OWB based in part on duration of time since illness and percentile scores on well-being and disability measures. They reported differential profiles of having attained lives that met their OWB definition based on previous illness profiles and comorbidities. They concluded that OWB, rather than solely symptom remission was an attainable goal for a proportion of their study participants. Our findings echo these results in the context of our study participants and their experience of a life worth living and beyond including flourishing. The very long nature of our interviews following treatment completion (up to 10 + years) is likely related to the finding of lives characterized by flourishing amongst our study participants, as they described needing considerable time and treatment to become fully well after long and complex illness histories. Currently there is limited knowledge of recovery amongst this population [12], which may be due to insufficient duration of recovery at post-treatment interview or evaluation [13].

A proposed theoretical model defining the wellness process from symptom remission to a life characterized as beyond health and wellness (flourishing) was developed from interview data and thematic analysis in the current study. This proposed model depicts the formulation of similarities and differences between symptom remission recovery, a life worth living, health and current life functioning consistent with accepted definitions of flourishing [18, 19].

### Transferability

As described earlier, our study participants were recruited from a self-harm and eating disorder organization (SHEDO) comprised of individuals with lived experience or interest therein. It is important to interpret our findings in terms of being transferable to those who identify as having recovered for a minimum of two years and who participate in organizations for individuals with lived experience. Further, the study was conducted in Sweden, which has a unique and fully socialized mental health system. Results from a meta-analysis comparing Swedish and German treatment studies indicated that novel and adapted programs were more effective in Sweden than adopted programs without any adaptation [27]. This an important transferability consideration with respect to treatment duration. Several participants emphasized the helpfulness of long treatments due to historically complex needs. Accordingly, our findings may not be directly applicable in settings that use the standard one-year outpatient DBT format [3].

All study participants identified as female, with predominantly non-heterosexual orientations. It is therefore unknown how well our findings may be appliable to individuals with other sexes, gender identities and sexual identities.

A strength of recruiting recovered individuals through SHEDO is that it might otherwise be quite difficult to locate and have contact with individuals this long (10 + years) after treatment. Therefore, we propose that SHEDO and similar organizations with large networks play a vital role for understanding life after treatment, as they create and maintain networks and communities that may otherwise be lost.

## Conclusion

Our interviews with individuals following treatment completion over time explored definitions, similarities, and differences among the constructs of symptom remission, recovery, a life worth living and flourishing from the perspective of those with lived experience of BPD and co-occurring clinical syndromes. Thematic analysis suggested that discovering oneself in relation to loved ones and in finding what sparks joy were key factors in becoming well. Our findings indicated overlapping stages in a wellness process described by participants as lengthy and non-linear, requiring long-term treatment and many years of follow-up. Our proposed theoretical pathway to wellness may support individuals diagnosed with BPD, their loved ones, and mental health professionals, providing hope for what is possible in the future.

Future research scaling the definitions and formulations of recovery, a life worth living and flourishing is required to generate operational definitions for each of these constructs. The development of treatment modules offering tools for fostering well-being and developing desired lives could support individuals to shorten their time from symptom remission into one or more stages of the wellness process associated with cultivating a life experienced as worth living and beyond.

### Electronic supplementary material

Below is the link to the electronic supplementary material.

Supplementary Material 1: Life Worth Living Interview Guide: Recovered Individuals interview Questions for LWL+ study

## Data Availability

The datasets generated and/or analysed during the current study are not publicly available due to the fact that they contain personal health information and are protected by laws that govern protection of personal health information. All data requests to the corresponding author would first be discussed amongst the research group and then vetted by a representative of the Swedish Ethical Review Authority.

## Footnotes

1 Backwards compatibility of the NVivo 1.6.1 was resolved through saving the NVivo project as a REFI-QDA Project so that the NVivo files could be compared.

2 The first author is a DBT therapist and researcher with years of experience in both roles, while the second author is a junior medical student who has not yet selected her theoretical orientation, nor has the third author, who is in the licensing year of clinical psychologists’ training. The last author is a senior psychiatrist and professor of Suicidology with an eclectic orientation and significant clinical experience of assessment and treatment of suicidal patients with various diagnostic profiles.

## List of abbreviations

BPD: Borderline personality disorder
DBT: Dialectical Behavior Therapy
PTSD: Posttraumatic Stress Disorder
RCT: Randomized controlled trial
