# Supplementary material for: Life experienced as worth living and beyond: a qualitative study of the pathways to recovery and flourishing amongst individuals treated for borderline personality disorder
Source: BMC Psychiatry. 2023 Nov 14;23:838. doi: 10.1186/s12888-023-05357-9 (PMC10644482; doi:10.1186/s12888-023-05357-9)
Supplement: Supplementary file 1 — Supplementary Material 1: Life Worth Living Interview Guide: Recovered Individuals interview Questions for LWL+ study [file 12888_2023_5357_MOESM1_ESM.docx]

## Life Worth Living Interview Guide: Recovered Individuals interview Questions for LWL+ study

1. a. Do you identify as “recovered” or do you think of your current life in a different way?
 *Please describe*

b. How long (years) have you felt this way?

2. a. What does your life look like today?

b. Do you experience life as "worth living" or meaningful? If so, in what way?

c. Do you experience your life as exciting or fun? If so, in what way?

d. Do you experience life as beautiful or do you feel that you are "in love" with your life? If so, in
 what way?

IF yes to 2b, c, d:

3. a. How did you go about creating a life you feel is worth living?

b. How did you go about creating a life you find exciting or fun?

c. How did you go about creating a life that is beautiful, or a life with which you feel “in love”?

4. Is having a life you feel is "worth living" the same as being recovered? Why or why not?

5. What are your happiest moments?

6. What are your most meaningful or fulfilling moments?

7. What are the most beautiful or enchanting moments about which you may feel “in love”?

8. Is there anything else I have not asked you about that you think is important to add?

9. Was your identity affected by getting sick? If so, in what way?

10. Did getting healthy affect your identity? If so, in what way?
